# Supplementary figures and images for: Pseudomonas syringae pv. tomato and the fall armyworm modulate the morpho-physiology and the metabolome of potato plants
Source: PLoS One. 2025 Dec 26;20(12):e0324111. doi: 10.1371/journal.pone.0324111 (PMC12742801; doi:10.1371/journal.pone.0324111)

A


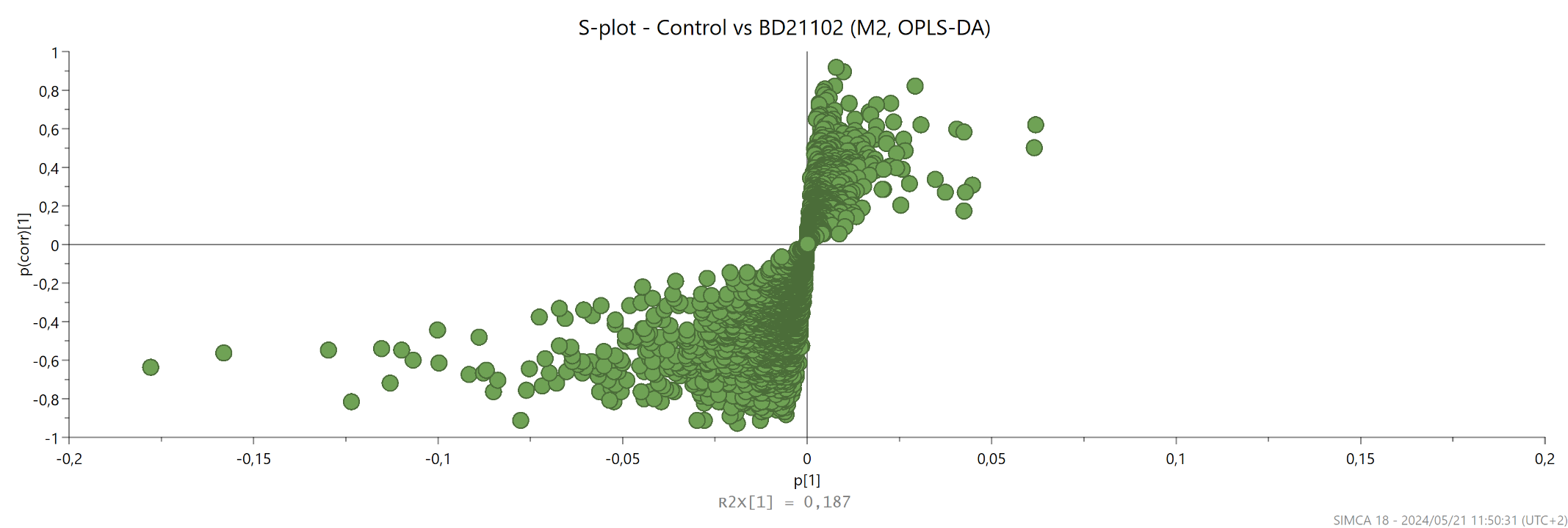


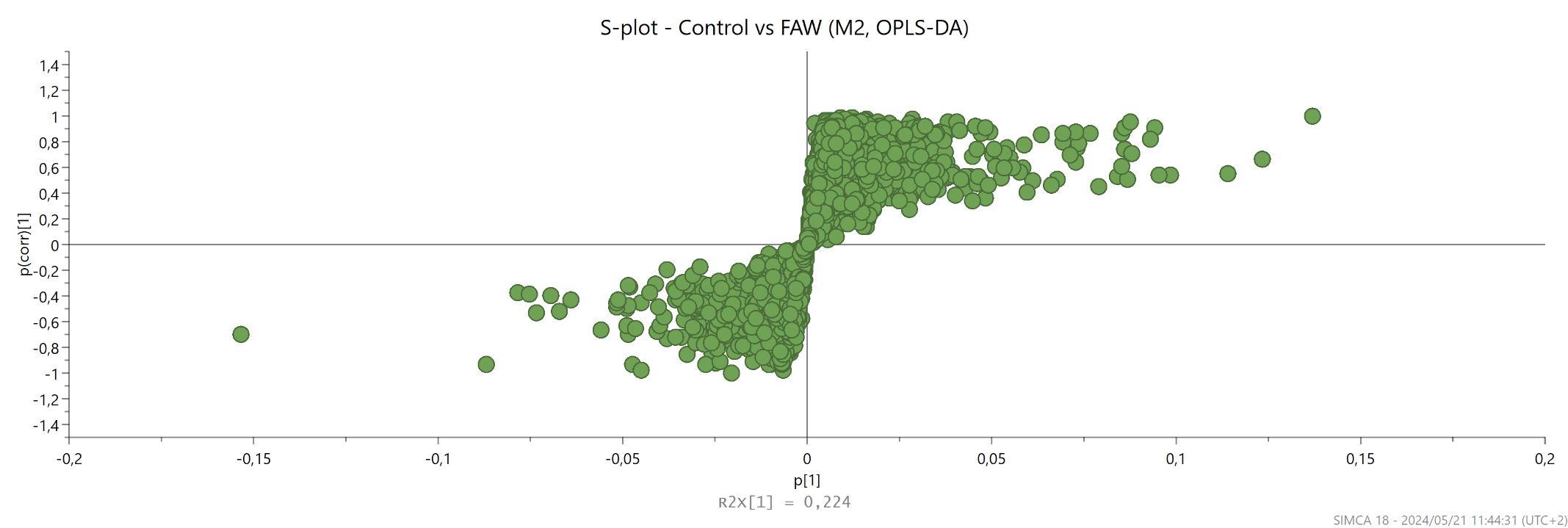


B


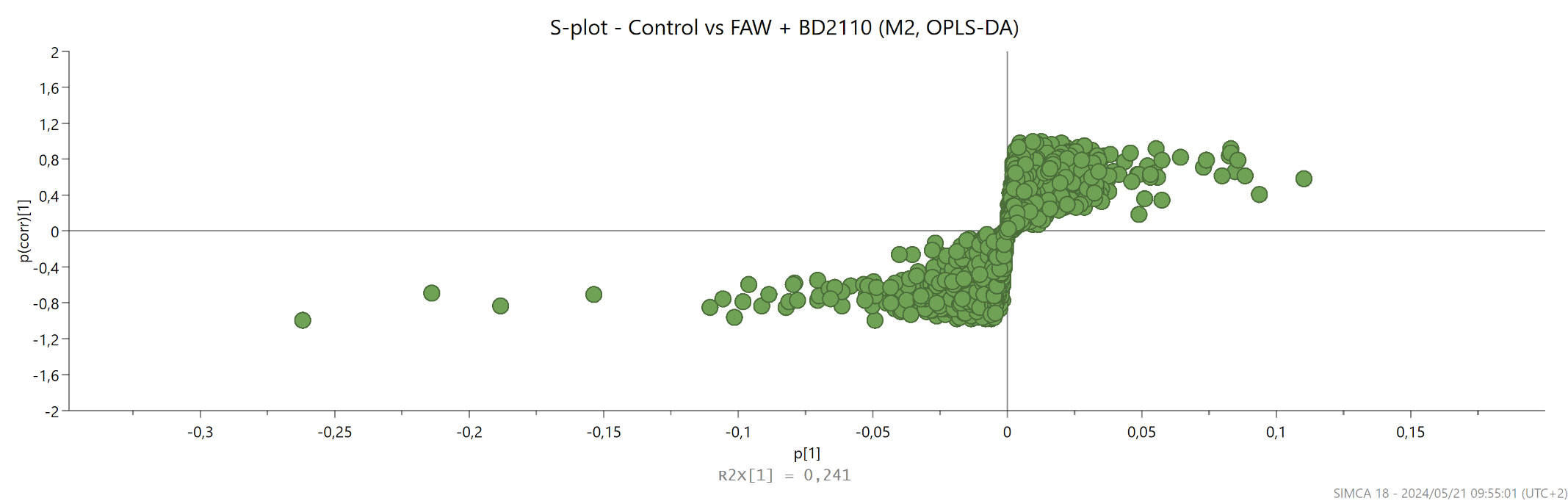


C

Supplementary fig 1.

Supplement: S1 Fig — An OPLS-DA S-plot utilizing Pareto scaling with mean centering to compare control and inoculated potato leaves. The UHPLC-qTOF-MS (Negative mode) data sets of potato leaf samples compared to control, BD2110, FAW, and BD2110 + FAW samples form the basis of the models. A (Control vs BD2110) B (Control vs FAW) C (Control vs FAW + BD2110). (DOCX) [file pone.0324111.s001.docx]

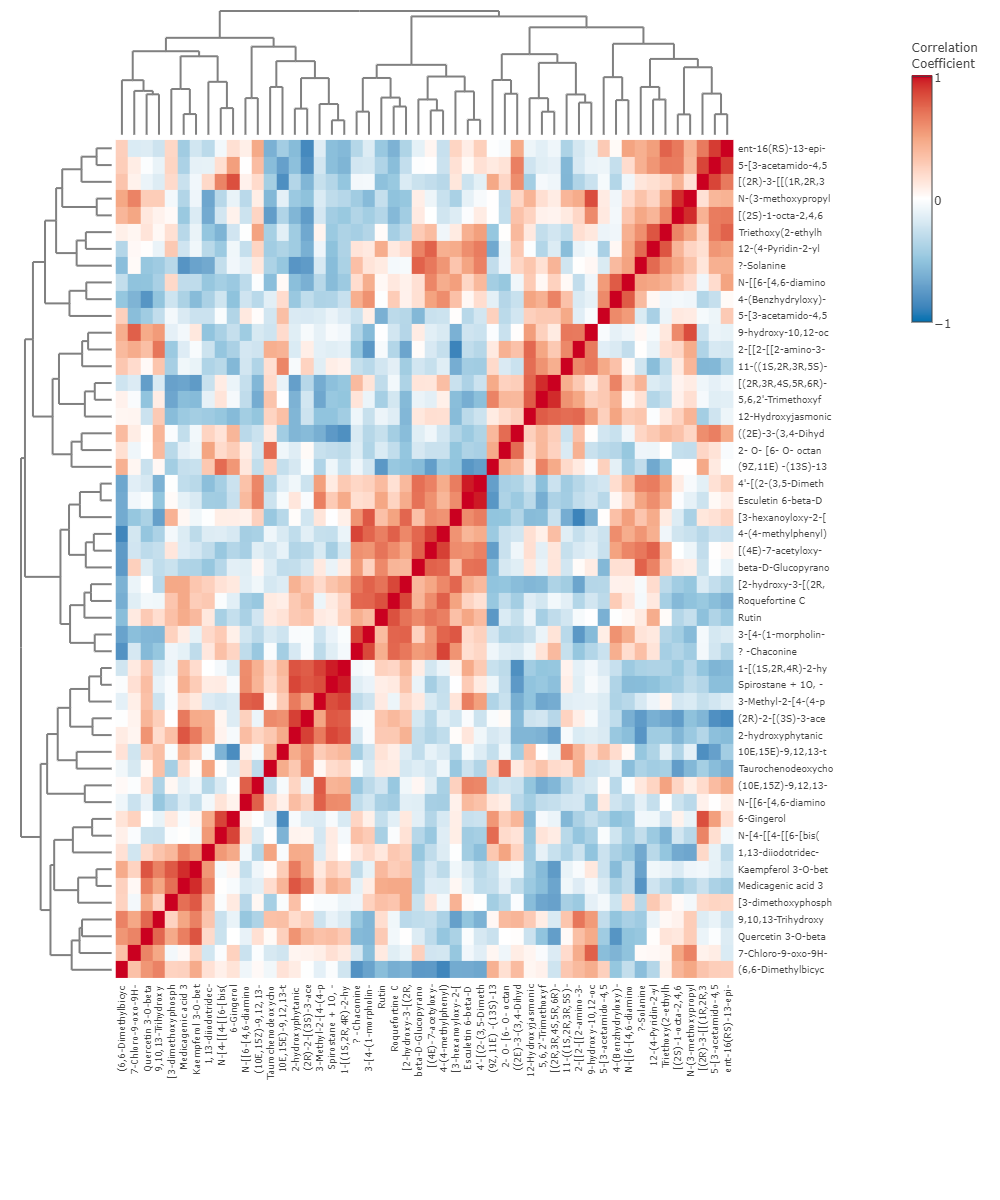


Heat Map Correlation Control vs BD2110

Supplementary fig 2.

Supplement: S2 Fig — The treatments were P. syringae pv. tomato, strain BD2110 against the untreated control. (DOCX) [file pone.0324111.s002.docx]

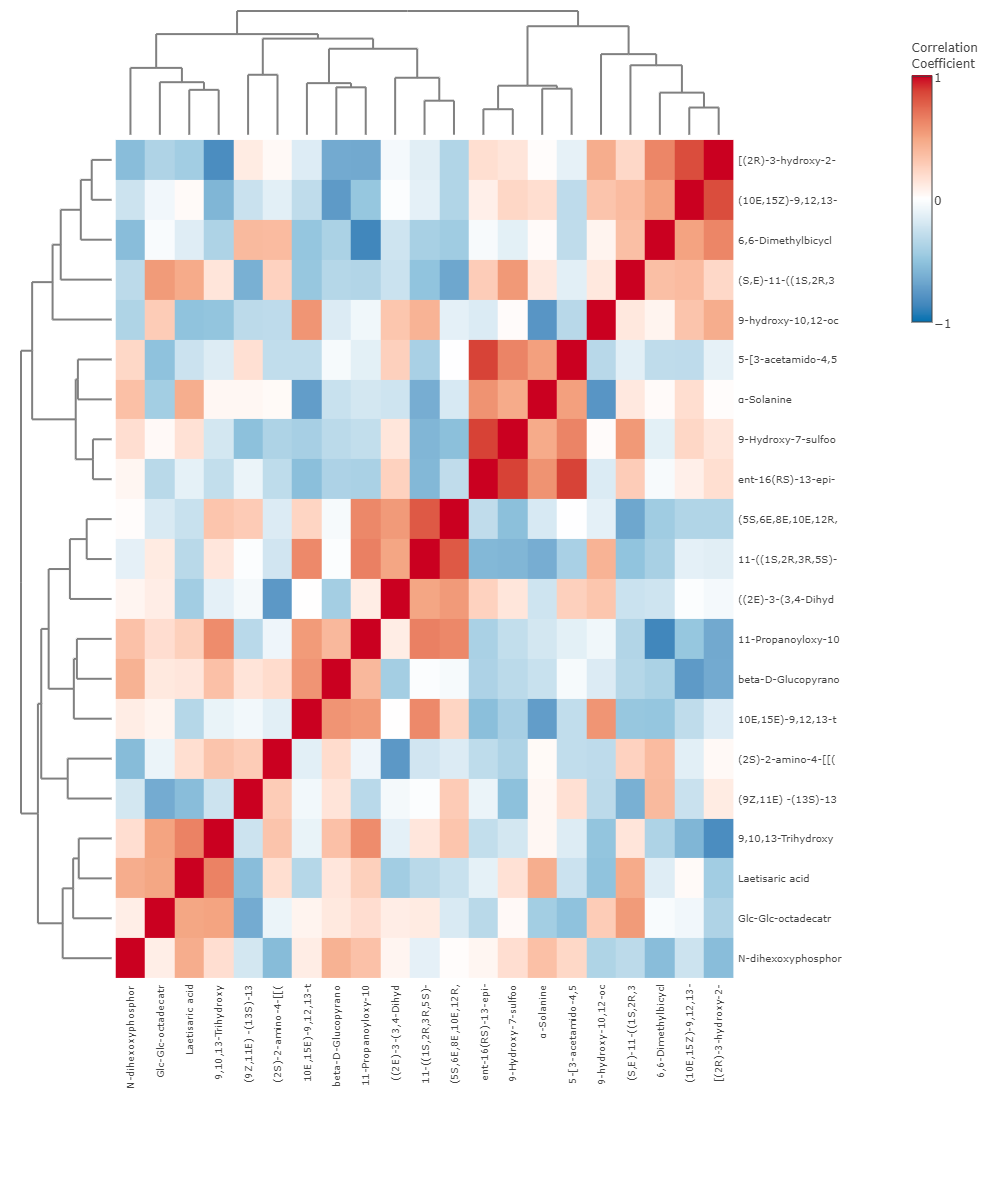


Heat Map Correlation Control vs FAW

Supplementary fig 3.

Supplement: S3 Fig — The treatments were against the untreated control. (DOCX) [file pone.0324111.s003.docx]

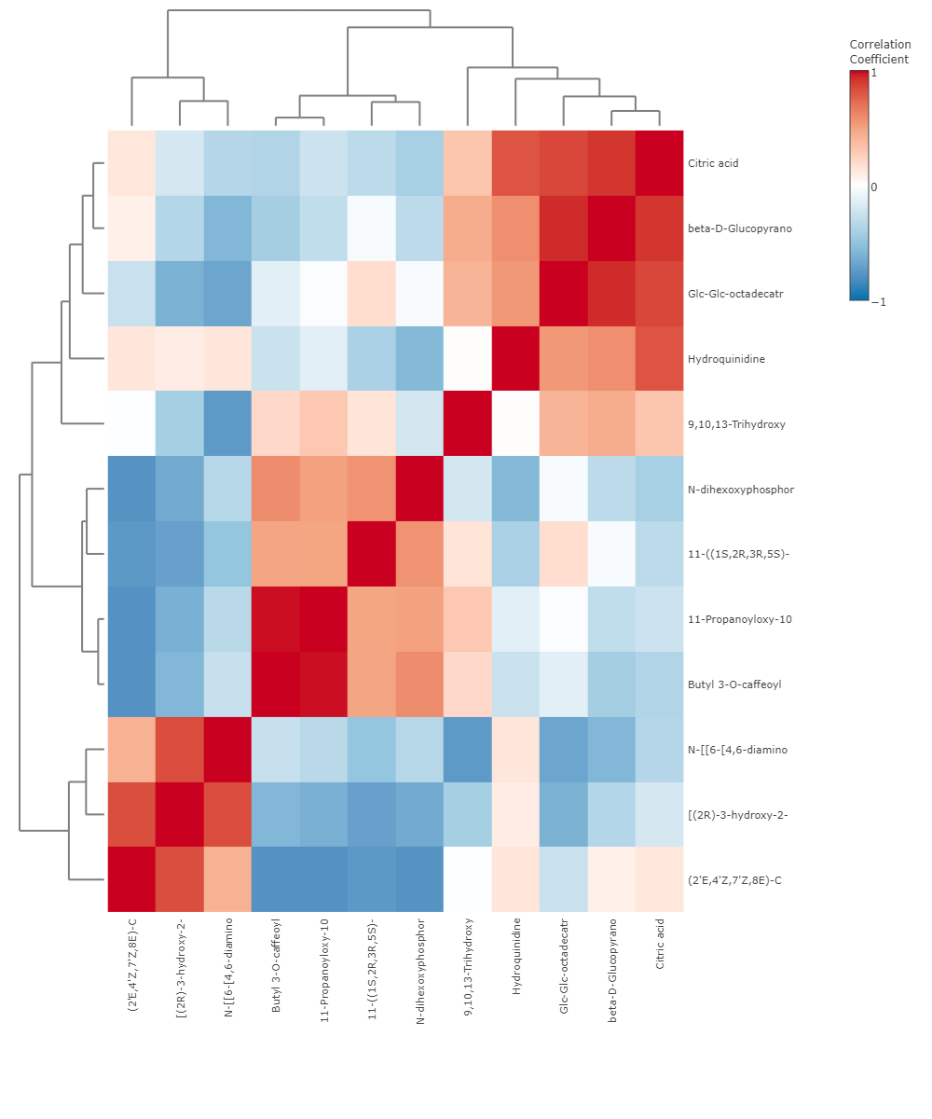


Heat Map Correlation Control vs BD2110 +FAW

Supplementary fig 4.

Supplement: S4 Fig — The treatments were P. syringae pv. tomato, strain BD2110 + FAW against the untreated control. (DOCX) [file pone.0324111.s004.docx]
