# Supplementary material for: Pseudomonas syringae pv. tomato and the fall armyworm modulate the morpho-physiology and the metabolome of potato plants
Source: PLoS One. 2025 Dec 26;20(12):e0324111. doi: 10.1371/journal.pone.0324111 (PMC12742801; doi:10.1371/journal.pone.0324111)
Supplement: S1 Table — The day of the infestation/inoculation was marked day 0 and plants were tracked throughout the 63 days of the experiment. Growth was measured at days 20, 27, and 34 and finally, the plants were harvested at day 63. The initial data was for tracking the plants and only data on days 34 and 63 was used to differentiate the treatments. (DOCX) [file pone.0324111.s005.docx]

|  | **Treatment/Insect** | **Treatment/Bacterium** | **Plant height** | **Stem diameter** | **Plant height** | **Stem diameter** | **Plant height** | **Stem diameter** | **Number of tubers** | **Total Tuber Weight** |
| --- | --- | --- | --- | --- | --- | --- | --- | --- | --- | --- |
| Units |  |  | cm | mm | cm | mm | cm | mm |  | g |
| Plant number | Day 0 | Day 0 | Day 20 | Day 20 | Day 27 | Day 27 | Day 34 | Day 34 | Day 63 | Day 63 |
| 1 | FAW | BD2110 | 40 | 5.35 | 40 | 5.35 | 40 | 5.35 | 13 | 109 |
| 2 | FAW | BD2110 | 76 | 7.86 | 81 | 8.08 | 81 | 8.08 | 12 | 223 |
| 3 | FAW | BD2110 | 58 | 5.92 | 60 | 7.9 | 63 | 7.9 | 8 | 184 |
| 4 | FAW | BD2110 | 47 | 5.11 | 54 | 5.81 | 59 | 5.81 | 9 | 121 |
| 5 | FAW | BD2110 | 55 | 5.1 | 56 | 6.13 | 60 | 6.27 | 11 | 201 |
| **Average** | | | 55.2 | 5.868 | 58.2 | 6.654 | 60.6 | 6.682 | 10.6 | 167.6 |
| 6 | FAW | No BD2110 | 50 | 3.86 | 52 | 4.04 | 54 | 5.64 | 6 | 115 |
| 7 | FAW | No BD2110 | 13 | 0.89 | 21 | 3.76 | 27 | 3.76 | 7 | 35 |
| 8 | FAW | No BD2110 | 34 | 6.53 | 34 | 6.53 | 35 | 6.53 | 6 | 130 |
| 9 | FAW | No BD2110 | 31 | 4.47 | 36 | 4.47 | 40 | 4.77 | 8 | 93 |
| **Average** | | | 32 | 3.9375 | 35.75 | 4.7 | 39 | 5.175 | 6.75 | 93.25 |
| 10 | No FAW | BD2110 | 54 | 6.99 | 55 | 6.99 | 56 | 6.99 | 6 | 150 |
| 11 | No FAW | BD2110 | 64 | 6.68 | 69 | 8.09 | 69 | 8.09 | 7 | 110 |
| 12 | No FAW | BD2110 | 54 | 4.6 | 60 | 5.85 | 63 | 5.85 | 13 | 138 |
| 13 | No FAW | BD2110 | 49 | 5.13 | 51 | 6.86 | 63 | 6.86 | 4 | 166 |
| **Average** | | | 55.25 | 5.85 | 58.75 | 6.9475 | 62.75 | 6.9475 | 7.5 | 141 |
| 14 | No FAW | No BD2110 | 40 | 5.25 | 40 | 5.25 | 56 | 5.25 | 7 | 78 |
| 15 | No FAW | No BD2110 | 55 | 6.86 | 60 | 6.86 | 65 | 6.86 | 4 | 187 |
| 16 | No FAW | No BD2110 | 60 | 6.62 | 76 | 6.62 | 80 | 7.11 | 9 | 159 |
| 17 | No FAW | No BD2110 | 57 | 6.6 | 60 | 6.6 | 62 | 7.34 | 8 | 127 |
| **Average** | | | 53 | 6.3325 | 59 | 6.3325 | 65.75 | 6.64 | 7 | 137.75 |

Supplementary table 1.
