# Supplementary material for: Pseudomonas syringae pv. tomato and the fall armyworm modulate the morpho-physiology and the metabolome of potato plants
Source: PLoS One. 2025 Dec 26;20(12):e0324111. doi: 10.1371/journal.pone.0324111 (PMC12742801; doi:10.1371/journal.pone.0324111)
Supplement: S2 Table — The physiological data was captured 13 days after infestation/inoculation. Two measurements were taken per plant. (DOCX) [file pone.0324111.s006.docx]

Supplementary table 2.

|  | **Treatment/Insect** | **Treatment/Bacterium** | **Photosynthetic rate** | **Stomatal conductance** | **Transpiration efficiency** | **Ci/Ca ratio** | **Water use efficiency** |
| --- | --- | --- | --- | --- | --- | --- | --- |
| Symbol |  |  | A | Gs | E | Ci/CaR | WUEi |
| Units |  |  | µmol(CO_2_)m^-2^.s^-1^ | Mol(H_2_O)m^-2^.s^-1^ | µmol(CO_2_) mol^-1^air^-1^ | µmol (CO_2_) m^-2^.s^-1^ | µmol (CO_2_) m^-1^.H_2_O |
| 1 | FAW | BD2110 | 5.935846954 | 0.145951476 | 4.925768704 | 0.720109516 | 40.67000291 |
| 1 | FAW | BD2110 | 5.948573864 | 0.146372003 | 4.938585697 | 0.720253341 | 40.64010709 |
| 2 | FAW | BD2110 | 7.716377734 | 0.112685204 | 4.348703869 | 0.564048354 | 68.4772928 |
| 2 | FAW | BD2110 | 7.701264979 | 0.11356276 | 4.377786172 | 0.567365556 | 67.81505658 |
| 3 | FAW | BD2110 | 7.643465396 | 0.24115704 | 5.855443654 | 0.777227293 | 31.69497098 |
| 3 | FAW | BD2110 | 7.889207043 | 0.241995262 | 5.886969184 | 0.771724081 | 32.60066743 |
| 4 | FAW | BD2110 | 5.462396212 | 0.092396764 | 3.134544845 | 0.621200737 | 59.11891288 |
| 4 | FAW | BD2110 | 5.501572182 | 0.092435137 | 3.134450422 | 0.619044583 | 59.51819159 |
| 5 | FAW | BD2110 | 2.468266098 | 0.032504126 | 1.233572701 | 0.534513407 | 75.93700888 |
| 5 | FAW | BD2110 | 2.499419575 | 0.032636799 | 1.23791065 | 0.530997696 | 76.58286436 |
| **Average** | | | 5.876639004 | 0.125169657 | 3.90737359 | 0.642648456 | 55.30550755 |
| 6 | FAW | No BD2110 | 3.451395308 | 0.049594675 | 1.641682196 | 0.570420681 | 69.59205383 |
| 6 | FAW | No BD2110 | 3.426210129 | 0.049996228 | 1.653832898 | 0.576127288 | 68.52937252 |
| 7 | FAW | No BD2110 | 9.155679495 | 0.141275187 | 4.368018107 | 0.577294768 | 64.80741369 |
| 7 | FAW | No BD2110 | 9.246012448 | 0.142493751 | 4.401411427 | 0.5765127 | 64.88714345 |
| 8 | FAW | No BD2110 | 6.319473792 | 0.161932228 | 4.280051768 | 0.733341062 | 39.02542362 |
| 8 | FAW | No BD2110 | 6.31048496 | 0.162245151 | 4.290057033 | 0.734065143 | 38.89475241 |
| 9 | FAW | No BD2110 | 6.869768675 | 0.117611027 | 3.769032038 | 0.624618097 | 58.41092344 |
| 9 | FAW | No BD2110 | 6.807591925 | 0.118563257 | 3.797496635 | 0.630092632 | 57.4173831 |
| **Average** | | | 6.448327092 | 0.117963938 | 3.525197763 | 0.627809046 | 57.69555826 |
| 10 | No FAW | BD2110 | 6.740552052 | 0.171784629 | 5.182806182 | 0.727058005 | 39.23838877 |
| 10 | No FAW | BD2110 | 7.226691122 | 0.172550079 | 5.20116081 | 0.711542753 | 41.88170283 |
| 11 | No FAW | BD2110 | 8.749059928 | 0.194862475 | 5.074953158 | 0.698156286 | 44.89863907 |
| 11 | No FAW | BD2110 | 8.743266182 | 0.196532891 | 5.097425385 | 0.700570926 | 44.48754681 |
| 12 | No FAW | BD2110 | 3.516126744 | 0.1091485 | 3.317021425 | 0.773117898 | 32.2141555 |
| 12 | No FAW | BD2110 | 3.592777347 | 0.109347042 | 3.324669681 | 0.769464338 | 32.85664864 |
| 13 | No FAW | BD2110 | 6.337658153 | 0.189030337 | 5.279221878 | 0.761504185 | 33.52720121 |
| 13 | No FAW | BD2110 | 6.33769572 | 0.189423149 | 5.288745124 | 0.761874565 | 33.45787322 |
| **Average** | | | 6.405478406 | 0.166584888 | 4.720750455 | 0.73791112 | 37.82026951 |
| 14 | No FAW | No BD2110 | 8.138210342 | 0.266675332 | 6.971285973 | 0.774803052 | 30.51729708 |
| 14 | No FAW | No BD2110 | 8.132847246 | 0.267617645 | 6.991179375 | 0.775514849 | 30.38980199 |
| 15 | No FAW | No BD2110 | 14.43616405 | 0.366272654 | 8.247817638 | 0.712608598 | 39.4137097 |
| 15 | No FAW | No BD2110 | 14.47714849 | 0.367997595 | 8.27495755 | 0.712887754 | 39.34033453 |
| 16 | No FAW | No BD2110 | 9.519848686 | 0.321893211 | 7.874486344 | 0.778414387 | 29.57455566 |
| 16 | No FAW | No BD2110 | 9.635262811 | 0.326175083 | 7.94693235 | 0.778497923 | 29.54015597 |
| 17 | No FAW | No BD2110 | 9.577402514 | 0.479414634 | 7.308482121 | 0.845308826 | 19.97728444 |
| 17 | No FAW | No BD2110 | 9.795831341 | 0.487437696 | 7.39760346 | 0.844163849 | 20.09658143 |
| **Average** | | | 10.46408944 | 0.360435481 | 7.626593101 | 0.777774905 | 29.8562151 |
