# Supplementary material for: Pseudomonas syringae pv. tomato and the fall armyworm modulate the morpho-physiology and the metabolome of potato plants
Source: PLoS One. 2025 Dec 26;20(12):e0324111. doi: 10.1371/journal.pone.0324111 (PMC12742801; doi:10.1371/journal.pone.0324111)
Supplement: S3 Table — The physiological data was captured 34 days after infestation/inoculation. Two measurements were taken per plant. (DOCX) [file pone.0324111.s007.docx]

Supplementary table 3.

|  | **Treatment/Insect** | **Treatment/Bacterium** | **Photosynthetic rate** | **Stomatal conductance** | **Transpiration efficiency** | **Ci/Ca ratio** | **Water use efficiency** |
| --- | --- | --- | --- | --- | --- | --- | --- |
| Symbol |  |  | A | Gs | E | Ci/CaR | WUEi |
| Units |  |  | µmol(CO_2_)m^-2^.s^-1^ | Mol(H_2_O)m^-2^.s^-1^ | µmol(CO_2_) mol^-1^air^-1^ | µmol (CO_2_) m^-2^.s^-1^ | µmol (CO_2_) m^-1^.H_2_O |
| 1 | FAW | BD2110 | 7.549163291 | 0.11959885 | 3.11743218 | 0.615735209 | 63.12070134 |
| 1 | FAW | BD2110 | 7.564679741 | 0.119978317 | 3.127874609 | 0.616044662 | 63.05039054 |
| 2 | FAW | BD2110 | 9.264207453 | 0.178322087 | 4.233544689 | 0.67179473 | 51.95210325 |
| 2 | FAW | BD2110 | 9.258096537 | 0.17869733 | 4.241011048 | 0.67257579 | 51.80881305 |
| 3 | FAW | BD2110 | 7.849718159 | 0.069992575 | 2.137526247 | 0.738317393 | 112.150727 |
| 3 | FAW | BD2110 | 7.826501311 | 0.069604435 | 2.124776739 | 0.738810445 | 112.4425663 |
| 4 | FAW | BD2110 | 8.052671073 | 0.164636214 | 4.195040994 | 0.687781391 | 48.91190644 |
| 4 | FAW | BD2110 | 8.010881552 | 0.164814226 | 4.198495406 | 0.689472585 | 48.60552235 |
| 5 | FAW | BD2110 | 7.613672188 | 0.112736526 | 3.256304711 | 0.589090688 | 67.53509685 |
| 5 | FAW | BD2110 | 7.722949753 | 0.114778329 | 3.310516723 | 0.590029493 | 67.28578341 |
| **Average** | | | 8.071254106 | 0.129315889 | 3.394252335 | 0.660965239 | 68.68636105 |
| 6 | FAW | No BD2110 | 7.876396301 | 0.167983552 | 3.914061795 | 0.703219302 | 46.88790177 |
| 6 | FAW | No BD2110 | 7.958473909 | 0.167872022 | 3.91207001 | 0.700306829 | 47.40798276 |
| 7 | FAW | No BD2110 | 9.039062947 | 0.187090305 | 4.595867002 | 0.689803701 | 48.31390346 |
| 7 | FAW | No BD2110 | 9.044613056 | 0.187462078 | 4.602763346 | 0.690175636 | 48.24769453 |
| 8 | FAW | No BD2110 | 5.128143899 | 0.238846028 | 5.482777919 | 0.842276776 | 21.47050104 |
| 8 | FAW | No BD2110 | 5.159674596 | 0.239731542 | 5.498829154 | 0.841968288 | 21.52271891 |
| 9 | FAW | No BD2110 | 6.740167707 | 0.047154298 | 1.492768747 | 0.648760107 | 142.9385645 |
| 9 | FAW | No BD2110 | 6.725569057 | 0.047459184 | 1.502308943 | 0.652295686 | 141.7126993 |
| **Average** | | | 7.209012684 | 0.160449876 | 3.875180865 | 0.721100791 | 64.81274579 |
| 10 | No FAW | BD2110 | 9.007842542 | 0.204805572 | 4.877302042 | 0.714092342 | 43.98240943 |
| 10 | No FAW | BD2110 | 9.094287289 | 0.206343978 | 4.910374238 | 0.713392224 | 44.07343204 |
| 11 | No FAW | BD2110 | 5.177058532 | 0.163299661 | 4.398129995 | 0.78327086 | 31.70281256 |
| 11 | No FAW | BD2110 | 5.196414609 | 0.163867235 | 4.412988945 | 0.78320794 | 31.71112652 |
| 12 | No FAW | BD2110 | 4.256839645 | 0.103691796 | 3.04207891 | 0.734139583 | 41.05281045 |
| 12 | No FAW | BD2110 | 4.303973177 | 0.103679803 | 3.043202549 | 0.731694464 | 41.51216601 |
| 13 | No FAW | BD2110 | 5.328222407 | 0.085532839 | 2.620554518 | 0.620517555 | 62.29446479 |
| 13 | No FAW | BD2110 | 5.341959359 | 0.085671876 | 2.623928762 | 0.620164905 | 62.35371049 |
| **Average** | | | 5.963324695 | 0.139611595 | 3.741069995 | 0.712559984 | 44.83536654 |
| 14 | No FAW | No BD2110 | 10.09668838 | 0.239182201 | 5.095344847 | 0.722948773 | 42.21337684 |
| 14 | No FAW | No BD2110 | 10.1252466 | 0.240443932 | 5.117985187 | 0.723412385 | 42.11063471 |
| 15 | No FAW | No BD2110 | 5.842373839 | 0.124551194 | 3.500056544 | 0.701771773 | 46.90740928 |
| 15 | No FAW | No BD2110 | 5.862878832 | 0.124877016 | 3.509018723 | 0.701554428 | 46.94922275 |
| 16 | No FAW | No BD2110 | 6.82274561 | 0.046470093 | 1.56152478 | 0.521744842 | 146.8201415 |
| 16 | No FAW | No BD2110 | 6.830526784 | 0.046726025 | 1.570112405 | 0.523179927 | 146.1824925 |
| 17 | No FAW | No BD2110 | 7.593679794 | 0.123154821 | 3.462555145 | 0.621184032 | 61.65962285 |
| 17 | No FAW | No BD2110 | 7.513491073 | 0.123073395 | 3.460063626 | 0.624556431 | 61.04886497 |
| **Average** | | | 7.585953864 | 0.133559835 | 3.409582657 | 0.642544074 | 74.23647067 |
