# Supplementary material for: Pseudomonas syringae pv. tomato and the fall armyworm modulate the morpho-physiology and the metabolome of potato plants
Source: PLoS One. 2025 Dec 26;20(12):e0324111. doi: 10.1371/journal.pone.0324111 (PMC12742801; doi:10.1371/journal.pone.0324111)
Supplement: S4 Table — (DOCX) [file pone.0324111.s008.docx]

Supplementary table 4.

| **Molecular Feature** | **Molecular formula** | **Proposed compound** | **m/z** | **Rt** | **Untreated** | **Treated** | **log2fold** | **VIP score** | **Adducts** |
| --- | --- | --- | --- | --- | --- | --- | --- | --- | --- |
|  |  |  |  | **min** |  |  |  |  |  |
| **BD2110** | | | | | | | | | |
| M327T6_6 | C_18_H_32_O_5_ | Epoxyoctadecane-dioic acid | 327.2746 | 6.35 | 16.87 | 18.67 | -1.79 | 3.34 | [M − H] ^−^ |
| M387T4_2 | C_18_H_28_O_9_ | 12-Hydroxyjasmonic acid glucoside | 387.1706 | 4.19 | 18.97 | 20.36 | -1.39 | 5.97 | [M − H] ^−^ |
| M388T4_2 | C_22_H_23_N_5_O_2_ | Roquefortine C | 388.1740 | 4.19 | 16.70 | 17.97 | -1.27 | 2.16 | [M − H] ^−^ |
| M989T7 | C_48_H_76_O_21_ | Medicagenic acid 3-O-triglucoside | 988.5292 | 6.91 | 16.88 | 18.03 | -1.15 | 1.62 | [M − H_2_O− H] − |
| M929T6_2 | C_45_H_72_O_17_ | Spirostane + 1O, -2H, O-Hex-dHex-dHex | 929.4867 | 6.45 | 17.09 | 18.24 | -1.15 | 1.84 | [M − H] ^−^ |
| M280T9_11 | C_19_H_23_NO | 4-(Benzhydryloxy)-1-methylpiperidine | 280.1775 | 9.23 | 15.72 | 17.69 | -1.98 | 3.24 | [M − H] ^−^ |
| M339T10 | C_15_H_16_O_9_ | Esculetin 6-beta-D-glucoside | 339.2037 | 10.44 | 19.18 | 19.96 | -0.78 | 2.13 | [M − H] ^−^ |
| M973T7 | C_15_H_32_O_8_ | (2R)-2-[(3S)-3-acetamido-2-oxopyrrolidin-1-yl]-3-hydroxy-N-[(3S,6S,9S,12S,15R,18S,21S)-6,9,18-tris(2-aminoethyl)-15-benzyl-3-[(1R)-1-hydroxyethyl]-12-(2-methylpropyl)-2,5,8,11,14,17,20-heptaoxo-1,4,7,10,13,16,19-heptazacyclotricos-21-yl]propenamide | 972.5295 | 6.93 | 17.27 | 18.30 | -1.03 | 1.63 | [M − H] ^−^ |
| M913T5_1 | C_45_H_73_NO_15_ | α-Solanine | 912.5081 | 5.47 | 19.68 | 20.64 | -0.96 | 4.42 | [M − H] ^−^ |
| M913T6_1 | C_45_H_73_NO_15_ | beta-D-Glucopyranoside, spirosol-5-en-3-yl O-6-deoxy-alpha-L-mannopyranosyl-(1->2)-O-[6-deoxy-alpha-L-mannopyranosyl-(1->4)]- | 912.5079 | 5.76 | 16.85 | 18.71 | -1.86 | 4.89 | M+FA-H |
| M578T9_1 | C_26_H_45_NO_9_S_2_ | Taurochenodeoxycholate-7-sulfate | 578.2791 | 8.78 | 17.30 | 18.40 | -1.10 | 2.05 | [M − H] ^−^ |
| M914T5_1 | C_47_H_79_O_15_P | [(2R)-3-{[(8Z,11Z,14Z)-5,6-dihydroxyicosa-8,11,14-trienoyl]oxy}-2-[(9Z,12Z,15Z)-octadeca-9,12,15-trienoyloxy]propoxy]({[(1S,2R,3R,4S,5S,6R)-2,3,4,5,6-pentahydroxycyclohexyl]oxy})phosphinate | 913.5108 | 5.48 | 18.92 | 19.84 | -0.92 | 3.23 | [M − H] ^−^ |
| M577T9_2 | C_24_H_42_N_4_O_12_ | N-[[6-[4,6-diamino-3-[[3-(aminomethyl)-5-hydroxy-6-(hydroxymethyl)-1,4-dioxan-2-yl]oxy]-2-hydroxycyclohexyl]oxy-3,4,5-trihydroxyoxan-2-yl]methyl]hex-5-ynamide | 577.2758 | 8.78 | 19.01 | 20.05 | -1.04 | 3.94 | [M − H] ^−^ |
| M311T9_1 | C_18_H_16_O_5_ | 5,6,2'-Trimethoxyflavone | 311.1722 | 9.22 | 19.69 | 20.38 | -0.68 | 4.54 | [M − H] ^−^ |
| M771T4_1 | C_33_H_40_O_21_ | Kaempferol 3-O-beta-D-glucosyl-(1->2)-beta-D-glucosyl-(1->2)-beta-D-glucoside | 771.2088 | 4.39 | 17.08 | 18.41 | -1.33 | 3.79 | [M − H] ^−^ |
| M343T6_3 | C_34_H_28_N_4_O_2_ | 12-(4-Pyridin-2-yltriazol-1-yl) dodecanoic acid | 343.2165 | 5.85 | 17.78 | 18.96 | -1.18 | 4.09 | [M − H] ^−^ |
| M407T10_2 | C_19_H_28_N_4_O_2_ | [2-hydroxy-3-[(2R,3S,4S,5R)-3,4,5-trihydroxy-6-(hydroxymethyl) oxan-2-yl] oxypropyl] decanoate | 407.2252 | 9.54 | 18.06 | 19.19 | -1.13 | 3.76 | [M − H] ^−^ |
| M265T9_6 | C_19_H_36_O_9_ | 7-Chloro-9-oxo-9H-indeno[1,2-b] pyrazine-2,3-dicarbonitrile | 265.2502 | 8.51 | 15.29 | 17.73 | -2.43 | 5.53 | [M − H] ^−^ |
| M325T10 | C_13_H_3_ClN_4_O | [(4E)-7-acetyloxy-6-hydroxy-2-methyl-10-oxo-2,3,6,7,8,9-hexahydrooxecin-3-yl] (E)-but-2-enoate | 325.1879 | 9.79 | 20.19 | 20.71 | -0.51 | 3.68 | [M − H] ^−^ |
| M433T9_3 | C_13_H_24_I_2_ | 1,13-diiodotridec-1-ene | 433.2412 | 9.46 | 18.48 | 19.53 | -1.05 | 4.10 | [M − H] ^−^ |
| M589T9_3 | C_37_H_60_N_4_O_3_ | N-[4-[[4-[[6-[bis(prop-2-enyl) amino]-6-oxohex-1-en-2-yl]amino]cyclohexyl]methyl]cyclohexyl]-N'-hepta-1,6-dien-4-ylpentanediamide | 589.3454 | 9.24 | 17.40 | 18.46 | -1.06 | 1.91 | [M − H_2_O− H] − |
| M625T4_1 | C_21_H_30_N_4_O_18_ | Quercetin 3-O-beta-D-glucosyl-(1->2)-beta-D-glucoside | 625.1489 | 4.45 | 17.33 | 18.59 | -1.26 | 3.88 | [M − H] ^−^ |
| M555T10_3 | C_28_H_50_N_2_O_6_S_2_ | 1-[(1S,2R,4R)-2-hydroxy-4,7,7-trimethyl-1-bicyclo [2.2.1]heptanyl]-N-[(1R,2R)-2-[[(1S,2R,4R)-2-hydroxy-4,7,7-trimethylbicyclo[2.2.1] heptanyl] methylsulfonylamino] cyclohexyl]methane sulfonamide | 555.2912 | 9.61 | 19.90 | 20.70 | -0.79 | 3.78 | [M − H_2_O− H] − |
| M579T9_2 | C_27_H_50_O_9_P_2_ | [3-dimethoxyphosphoryloxy-2-[(5Z,8Z,11Z,14Z)-icosa-5,8,11,14-tetraenoxy] propyl] dimethyl phosphate | 579.2917 | 9.30 | 17.92 | 18.93 | -1.02 | 3.28 | [M − H] ^−^ |
| M609T5_1 | C_34_H_22_N_6_O_6_ | Rutin | 609.1537 | 4.85 | 17.03 | 18.57 | -1.54 | 5.12 | [M − H] ^−^ |
| M293T8_15 | C_17_H_26_O_4_ | 6-Gingerol | 293.2154 | 8.44 | 17.51 | 18.82 | -1.31 | 5.98 | [M − H] ^−^ |
| M556T10_2 | C_23_H_40_O_15_ | 2-*O*-[6-*O*-octanoyl-α-D-glucosyl-(1→6)-α-D-glucosyl]-D-glycerate | 556.2946 | 9.62 | 18.15 | 18.92 | -0.77 | 2.05 | [M − H] ^−^ |
| M431T9_2 | C_21_H_38_O_10_ | [3-hexanoyloxy-2-[(2R,3R,4S,5S,6R)-3,4,5-trihydroxy-6-(hydroxymethyl) oxan-2-yl] oxypropyl] hexanoate | 431.2255 | 9.00 | 17.75 | 18.92 | -1.17 | 3.77 | [M − H_2_O− H] − |
| M329T7_2 | C_18_H_34_O_5_ | 9,10,13-Trihydroxyoctadec-11-enoic acid | 329.2375 | 6.65 | 21.64 | 22.39 | -0.75 | 7.65 | [M − H] − |
| M851T6_1 | C_45_H_73_NO_14_ | α -Chaconine | 850.5069 | 5.51 | 18.32 | 18.96 | -0.64 | 2.02 | [M − H] − |
| M329T4_1 | C_19_H_29_NO_3_ | 3-Methyl-2-[4-(4-propan-2-ylphenyl)butanoylamino]pentanoic acid | 329.1280 | 4.42 | 18.33 | 19.05 | -0.72 | 2.26 | [M − H_2_O− H] − |
| M327T6_3 | C_18_H_32_O_5_ | (10E,15Z)-9,12,13-Trihydroxy-10,15-octadecadienoic acid | 327.2215 | 6.28 | 21.73 | 22.38 | -0.65 | 4.88 | [M − H] − |
| M345T6_2 | C_19_H_30_N_4_O_2_ | 3-[4-(1-morpholin-4-ylethyl)piperidin-1-yl]-N-pyridin-3-ylpropanamide | 345.2316 | 6.13 | 15.10 | 17.77 | -2.67 | 6.17 | [M − H] − |
| M494T6_22 | C_19_H_35_N_3_O_9_ | 5-[3-acetamido-4,5-dihydroxy-6-(hydroxymethyl) oxan-2-yl]oxy-N-[3-(3-hydroxypropanoylamino)propyl]pentanamide | 494.2385 | 5.99 | 19.00 | 19.68 | -0.67 | 3.26 | [M + CH_2_O− H] − |
| M449T5_2 | C_20_H_28_N_4_O_5_ | 2-[[2-[[2-amino-3-(1H-indol-3-yl) propanoyl]amino]-3-methylbutanoyl]amino]-3-hydroxybutanoic acid | 449.2082 | 5.05 | 19.01 | 19.73 | -0.73 | 4.01 | [M − H_2_O− H] − |
| M930T6_1 | C_30_H_42_N_7_O_19_P_3_S | ((2E)-3-(3,4-Dihydroxyphenyl) prop-2-enoyl-CoA | 929.5055 | 5.63 | 16.00 | 18.06 | -2.07 | 5.15 | [M − H] − |
| M509T5_3 | C_26_H_39_O_8_P | [(2S)-1-octa-2,4,6-triynoyloxy-3-phosphonooxypropan-2-yl] pentadecanoate | 509.2302 | 4.90 | 18.60 | 19.50 | -0.90 | 4.13 | [M − H] − |
| M447T6_2 | C_27_H_32_N_2_O_4_ | 4-(4-methylphenyl)-N-[1-[4-(4-methylphenyl)-4-oxobutanoyl] piperidin-4-yl]-4-oxobutanamide | 447.2290 | 5.98 | 18.81 | 19.43 | -0.61 | 3.19 | [M − H] − |
| M493T6_3 | C_21_H_36_O_10_ | (6,6-Dimethylbicyclo [3.1.1] hept-2-yl)methyl 6-O-[(2R,3R,4R)-3,4-dihydroxy-4-(hydroxymethyl)tetrahydro-2-furanyl]-beta-D-glucopyranoside | 493.2353 | 5.99 | 20.87 | 21.47 | -0.60 | 6.59 | [M − H] − |
| M433T2 | C_18_H_28_O_13_ | [(2R,3R,4S,5R,6R)-3,4-diacetyloxy-6-hydroxy-5-[(2S,3R,4S,5S,6R)-3,4,5-trihydroxy-6-methyloxan-2-yl] oxyoxan-2-yl]methyl acetate | 433.1376 | 2.14 | 19.35 | 20.01 | -0.66 | 4.08 | [M − H] − |
| M293T8_11 | C_15_H_34_O_3_S | Triethoxy(2-ethylheptyl)-lambda4-sulfane | 293.1797 | 7.80 | 17.05 | 20.52 | -3.47 | 14.92 | [M − H] − |
| M897T6_1 | [C_43_H_82_NO_17_P](https://pubchem.ncbi.nlm.nih.gov/#query=C43H82NO17P) | [(2R)-3-[[(1R,2R,3S,4R,5R,6R)-2-[(2R,3R,4R,5S,6R)-3-amino-4,5-dihydroxy-6-(hydroxymethyl)oxan-2-yl]oxy-3,4,5,6-tetrahydroxycyclohexyl]oxy-hydroxyphosphoryl]oxy-2-tetradecanoyloxypropyl] tetradecanoate | 896.5136 | 5.51 | 21.36 | 21.86 | -0.49 | 7.64 | [M -H2O− H] − |
| M433T5_4 | C_20_H_28_N_4_O_4_ | N-(3-methoxypropyl)-1-[2-(3-methyl-2-oxobenzimidazol-1-yl) acetyl] piperidine-4-carboxamide | 433.2129 | 4.66 | 20.66 | 21.19 | -0.53 | 5.20 | [M − H] − |
| M421T4_2 | C_26_H_22_N_4_O_2_ | 4'-[(2-(3,5-Dimethyl-pyrazol-1-yl)-benzimidazol-1-yl)-methyl] biphenyl-2-carboxylic acid | 421.1686 | 4.33 | 19.00 | 19.50 | -0.51 | 3.13 | M − H] − |
| M343T6_1 | C_18_H_32_O_6_ | ent-16(RS)-13-epi-ST-D14-9-PhytoF | 343.2165 | 5.59 | 19.20 | 19.77 | -0.56 | 2.40 | M − H] − |
| M295T8_28 | C_18_H_32_O_3_ | 9-hydroxy-10,12-octadecadienoic acid | 295.2313 | 8.08 | 20.31 | 20.71 | -0.39 | 7.20 | [M − H] − |
| M311T8_2 | C_18_H_32_O_4_ | (9Z,11E) -(13S)-13-Hydroperoxyoctadeca-9,11-dienoic acid | 311.2262 | 7.51 | 19.80 | 20.41 | -0.61 | 3.42 | [M − H] − |
| M677T6 | C_18_H_32_O_5_ | 10E,15E)-9,12,13-trihydroxyoctadeca-10,15-dienoic acid | 677.4329 | 6.35 | 20.57 | 20.92 | -0.36 | 7.62 | 2M-2H+Na |
| M327T6_9 | C_18_H_32_O_5_ | 11-((1S,2R,3R,5S)-2-ethyl-3,5-dihydroxycyclopentyl)-9R-hydroxyundec-10E-enoic acid | 327.3382 | 6.35 | 18.41 | 16.18 | 2.23 | 9.19 | [M − H] − |
| M494T6_21 | C_19_H_35_N_3_O_9_ | 5-[3-acetamido-4,5-dihydroxy-6-(hydroxymethyl) oxan-2-yl] oxy-N-[3-(3-hydroxypropanoylamino) propyl]pentanamide | 494.1957 | 5.98 | 18.96 | 17.41 | 1.55 | 9.07 | [M − H] − |
| **FAW** | | | | | | | | | |
| M327T6_3 | C_18_H_32_O_5_ | (10E,15Z)-9,12,13-Trihydroxy-10,15-octadecadienoic acid | 327.2216 | 6.29 | 22.03 | 22.26 | -0.23 | 11.12 | [M − H] − |
| M930T6_1 | C_30_H_42_N_7_O_19_P_3_S | ((2E)-3-(3,4-Dihydroxyphenyl) prop-2-enoyl-CoA | 929.5051 | 5.62 | 16.35 | 18.19 | -1.84 | 6.77 | [M − H] − |
| M913T5_1 | C_45_H_73_NO_15_ | α-Solanine | 912.5081 | 5.47 | 20.01 | 20.53 | -0.52 | 8.32 | [M − H] − |
| M913T6_1 | C_45_H_73_NO_15_ | beta-D-Glucopyranoside, spirosol-5-en-3-yl O-6-deoxy-alpha-L-mannopyranosyl-(1->2)-O-[6-deoxy-alpha-L-mannopyranosyl-(1->4)]- | 912.5078 | 5.75 | 18.13 | 19.86 | -1.73 | 12.43 | [M − H] − |
| M494T6_20 | C_19_H_35_N_3_O_9_ | 5-[3-acetamido-4,5-dihydroxy-6-(hydroxymethyl) oxan-2-yl] oxy-N-[3-(3-hydroxypropanoylamino) propyl] pentanamide | 494.2385 | 5.99 | 19.37 | 19.85 | -0.47 | 4.82 | [M − H] − |
| M493T6_3 | C_21_H_36_O_10_ | 6,6-Dimethylbicyclo [3.1.1]hept-2-yl) methyl 6-O-[(2R,3R,4R)-3,4-dihydroxy-4-(hydroxymethyl)tetrahydro-2-furanyl]-beta-D-glucopyranoside | 493.2352 | 5.99 | 21.24 | 21.53 | -0.29 | 6.93 | [M − H] − |
| M421T10 | C_20_H_38_O_9_ | [(2R)-3-hydroxy-2-[(2R,3R,4S,5R,6R)-3,4,5-trihydroxy-6-(hydroxymethyl) oxan-2-yl] oxpropyl] undecanoate | 421.2410 | 10.32 | 17.88 | 15.31 | 2.58 | 2.33 | [M − H] − |
| M341T6_1 | C_17_H_31_N_2_O_3_P | N-dihexoxyphosphorylpyridin-3-amine | 341.2009 | 5.96 | 19.35 | 17.82 | 1.54 | 5.61 | [M − H] − |
| M721T8 | C_33_H_56_O_14_ | Glc-Glc-octadecatrienoyl-sn-glycerol (isomer 2) (PUT) | 721.3741 | 7.99 | 19.03 | 17.36 | 1.68 | 2.53 | [M − H] − |
| M325T6_2 | C_18_H_30_O_5_ | (S,E)-11-((1S,2R,3R)-2-ethyl-3-hydroxy-5-oxocyclopentyl)-9-hydroxyundec-10-enoic acid | 325.2057 | 6.18 | 18.85 | 17.45 | 1.40 | 5.56 | [M − H] − |
| M327T6_5 | C_18_H_32_O_5_ | 11-((1S,2R,3R,5S)-2-ethyl-3,5-dihydroxycyclopentyl)-9R-hydroxyundec-10E-enoic acid | 327.2534 | 6.35 | 19.10 | 17.45 | 1.65 | 6.99 | [M − H] − |
| M343T6_2 | C_18_H_32_O_6_ | ent-16(RS)-13-epi-ST-D14-9-PhytoF | 343.2165 | 5.59 | 19.59 | 18.75 | 0.84 | 4.24 | M − H] − |
| M311T8_2 | C_18_H_32_O_4_ | (9Z,11E) -(13S)-13-Hydroperoxyoctadeca-9,11-dienoic acid | 311.2262 | 7.51 | 20.20 | 18.83 | 1.36 | 9.09 | M − H] − |
| M296T8_37 | C_18_H_32_O_3_ | Laetisaric acid | 296.2346 | 8.08 | 18.80 | 17.98 | 0.81 | 2.54 | [M − H] − |
| M191T1_2 | C_6_H_8_O_7_ | Citric acid | 191.0582 | 1.13 | 20.79 | 19.84 | 0.95 | 6.54 | [M − H] − |
| M397T7 | C_21_H_36_O_8_ | 11-Propanoyloxy-10-(1-propanoyloxyethoxycarbonyl) dodecanoic acid | 397.2253 | 6.65 | 19.77 | 19.66 | 0.11 | 4.62 | [M − H_2_O− H] − |
| M329T7_2 | C_18_H_34_O_5_ | 9,10,13-Trihydroxyoctadec-11-enoic acid | 329.2375 | 6.65 | 22.03 | 21.83 | 0.20 | 12.74 | [M − H] − |
| M363T8_3 | C_21_H_32_O_5_ | (5S,6E,8E,10E,12R,14E)-5,12-dihydroxy-21-oxohenicosa-6,8,10,14-tetraenoic acid | 363.2194 | 8.08 | 18.76 | 18.13 | 0.63 | 2.16 | [M − H] − |
| M361T8_1 | C_18_H_36_O_6_S | 9-Hydroxy-7-sulfooctadecanoic acid | 361.2037 | 7.79 | 19.85 | 19.57 | 0.28 | 3.52 | [M − H] − |
| M677T6 | C_18_H_32_O_5_ | 10E,15E)-9,12,13-trihydroxyoctadeca-10,15-dienoic acid | 677.4329 | 6.35 | 20.98 | 20.63 | 0.35 | 6.75 | [2M-2H+Na] |
| M295T8_27 | C_18_H_32_O_3_ | 9-hydroxy-10,12-octadecadienoic acid | 295.2312 | 8.08 | 20.70 | 20.16 | 0.54 | 4.29 | [M − H] − |
| **FAW+BD2110** | | | | | | | | | |
| M913T6_1 | C_45_H_73_NO_15_ | beta-D-Glucopyranoside, spirosol-5-en-3-yl O-6-deoxy-alpha-L-mannopyranosyl-(1->2)-O-[6-deoxy-alpha-L-mannopyranosyl-(1->4)]- | 912.5078 | 5.67 | 18.69 | 21.58 | -2.89 | 11.44 | [M − H] − |
| M329T7_2 | C_18_H_34_O_5_ | 9,10,13-Trihydroxyoctadec-11-enoic acid | 329.2374 | 6.65 | 22.03 | 22.52 | -0.49 | 9.41 | [M − H] − |
| M327T6_9 | C_18_H_32_O_5_ | 11-((1S,2R,3R,5S)-2-ethyl-3,5-dihydroxycyclopentyl)-9R-hydroxyundec-10E-enoic acid | 327.3342 | 6.34 | 18.86 | 17.16 | 1.70 | 4.87 | [M − H] − |
| M721T8 | C_33_H_56_O_14_ | Glc-Glc-octadecatrienoyl-sn-glycerol (isomer 2) (PUT) | 721.3740 | 7.99 | 19.03 | 17.81 | 1.22 | 2.72 | [M − H] − |
| M421T10 | C_20_H_38_O_9_ | [(2R)-3-hydroxy-2-[(2R,3R,4S,5R,6R)-3,4,5-trihydroxy-6-(hydroxymethyl) oxan-2-yl] ox propyl] undecanoate | 421.2409 | 10.32 | 17.88 | 16.45 | 1.43 | 3.98 | [M − H] − |
| M341T6_1 | C_17_H_31_N_2_O_3_P | N-dihexoxyphosphorylpyridin-3-amine | 341.2008 | 5.97 | 19.35 | 18.56 | 0.80 | 3.90 | [M − H] − |
| M325T6 | C_20_H_26_N_2_O_2_ | Hydroquinidine | 325.2056 | 6.28 | 18.85 | 18.10 | 0.75 | 5.15 | [M − H] − |
| M191T1_5 | C_6_H_8_O_7_ | Citric acid | 191.1150 | 1.13 | 19.62 | 18.63 | 0.99 | 6.69 | [M − H] − |
| M433T5_2 | C_20_H_26_O9 | Butyl 3-O-caffeoylquinate | 433.2127 | 4.65 | 20.98 | 20.64 | 0.33 | 7.04 | [M − H] − |
| M577T9_1 | C_24_H_42_N_4_O_12_ | N-[[6-[4,6-diamino-3-[[3-(aminomethyl)-5-hydroxy-6-(hydroxymethyl)-1,4-dioxan-2-yl]oxy]-2-hydroxycyclohexyl]oxy-3,4,5-trihydroxyoxan-2-yl]methyl]hex-5-ynamide | 577.2757 | 8.79 | 19.41 | 18.96 | 0.45 | 4.82 | [M − H] − |
| M397T7 | C_21_H_36_O_8_ | 11-Propanoyloxy-10-(1-propanoyloxyethoxycarbonyl) dodecanoic acid | 397.2252 | 6.66 | 19.77 | 19.40 | 0.37 | 2.28 | [M − H_2_O− H] − |
| M291T8_1 | C_18_H_28_O_3_ | **(2'E,4'Z,7'Z,8E)-Colnelenic acid** | 291.1998 | 7.86 | 20.11 | 19.87 | 0.24 | 2.59 | [M − H] − |

Analysed using high-definition MS in UHPLC negative mode, this summary presents the annotated metabolites found in potato from **BD2110, FAW** and **FAW+BD2110** treated leaf respectively. The metabolites that played a role in the distinguishing differences in the changed metabolomes were determined using OPLS-DA S plots, with a threshold score values 0.05. Annotations are associated with the detection of metabolites using MS2. The concentrations of metabolites are scaled using a logarithmic base of 2. The metabolites shown are the ones that exhibit differences between the treated and untreated samples.
