# Supplementary material for: Pseudomonas syringae pv. tomato and the fall armyworm modulate the morpho-physiology and the metabolome of potato plants
Source: PLoS One. 2025 Dec 26;20(12):e0324111. doi: 10.1371/journal.pone.0324111 (PMC12742801; doi:10.1371/journal.pone.0324111)
Supplement: Attachment — Submitted filename: Response to Reviewers.docx [file pone.0324111.s011.docx]

Prof Khayalethu Ntushelo

Room 326B, Calabash Building, Science Campus, c/n Christiaan De Wet and Pioneer Avenue, Florida, 1710

E-mail	:	ntushk@unisa.ac.za

To	:	The Editor, PLoS One

Date	:	3 August 2025

**SUBJECT: Corrections on the manuscript PONE-D-25-21507**

Dear Editor

I am pleased to learn that both reviewers recommend the article for publication. I have reworked it to incorporate their suggestions. Additionally, I have scrutinized all sorts all issues. I thank you for the work you have done on this manuscript. The suggestions were valuable, and the authors considered and incorporated them. Additions in the corrected manuscript appear in red font (for reviewer 1) and blue font (for reviewer 2). I have loaded both corrected documents but they are not track-changed. The suggestions and the actions taken are listed below. The suggestions appear in red and the responses in black. I have included both the concerns of Reviewer 1 and Reviewer 2. Before I began with the suggestions of the reviewers I wished to address the general concerns raised by the editorial staff.

**General matters**

Style requirements and file naming.

These matters have been addressed.

Financial disclosure

This financial disclosure statement was added “This study was partially funded by the National Research Foundation of South Africa (www.nrf.ac.za) under the grant TTK170413227119. The funds were allocated to Khayalethu Ntushelo and this research was covered solely by his funds. The funders had no role in study design, data collection and analysis, decision to publish, or preparation of the manuscript.

Captions for supporting information files

Captions for figures, tables and supplementary material are added at the end of the manuscript just before the reference list.

Submission of data

Data on the growth, number of tubers, total tuber weight, leaf gas exchange (physiological) parameters, and metabolites has been submitted as requested. We have further submitted screenshots of the data analysis process so that our methods can be scrutinized. Please note that for the metabolite data we were forced to have equal blocks of the treatments unlike as in the growth and physiology data.

Submission of figure files in PACE

All figures have been submitted as requested.

**Reviewer 1**

The manuscript states that uninoculated plants were mock-inoculated with buffer, but it does not clarify whether the buffer infiltration itself caused any physiological or metabolomic effects.

The bacteria were suspended in an inoculation buffer. This same buffer was used to mock-inoculate the control plants so that only the effects of the bacterium could be tested and observed. I have now added the word “inoculation” to show it was the inoculation buffer that was used to cancel its effect in the experimental plants. I have also added the sentence “Mock inoculation with the buffer was meant to cancel the effects of the buffer in the bacterial culture”.

The use of five first-instar FAW larvae per plant is noted, but there is no information on how larval feeding was controlled or monitored (e.g., whether larvae were replaced if they died or stopped feeding, or how feeding damage was quantified).

Five first instar larvae per plant were administered once at the beginning, day 0 as per the timeline (Figure 2). The effect of the larval feeding was measured indirectly by measuring growth and physiological indices. It turned out that the pest supressed the physiological indices, the rate of photosynthesis, stomatal conductance, transpiration efficiency and the ratio of intercellular to ambient dioxide concentration 13 days after infestation. The lowering of growth by the pest could be resulting from this physiological drag which, however, dampened 34 days after infestation. Total tuber weight was affected by the FAW. On the larvae, they were not replaced and they displayed unfitness on the potato plant and this was shown by limited feeding and their disappearance 13 days (first plant examination) post infestation. We could only speculate that they had pupated by day 13 post infestation. It is known that potato is not a preferred host of the FAW as demonstrated by Guo et al. 2021 who showed that compared to maize the FAW survives less on potato. I have attached photos (not to be included for publication) of the potato plants taken 47 days post infestation and these photos demonstrate minimal and inconsistent feeding. The leaves were still green with minimal damage which reflected limited nibbling rather than feeding on a preferred host. Despite the lower that expected feeding on the leaves, the pest still reduced plant growth, modulated the physiology of the plant and caused metabolomic reprogramming of the treated plants.

The metabolomic analysis identifies 50, 22, and 34 metabolites affected in Pst, FAW, and combined treatments, respectively, but the manuscript lacks details on how these metabolites were selected or validated. For example, were they identified based on statistical significance, fold change thresholds, or biological relevance?

Only metabolites with VIP scores greater than one were selected. The higher the VIP score the more the metabolite contributes to the chemometric projection. Although of significance, the log2fold change was not used for selecting the metabolites to be included. We understand that the greater the deviation of the log2fold change from zero the bigger the response of the metabolite to the experimental treatment. The corrected manuscript has this explanation written in red “Only metabolites with VIP scores greater than one were selected for inclusion on Supplementary table 1. The higher the VIP score the more the metabolite contributes to the chemometric projection. Although of significance, the log2fold change was not used for selecting the metabolites to be included”. We further selected a few metabolites for discussion, and the selection was based on relevance to the current study. We selected rutin, α-solanine, 6-gingerol, laetisaric acid and citric acid.

The abstract is dense and includes technical details (e.g., specific correlations) that may overwhelm readers. It should focus on key findings and their implications. Emphasizing the interaction between Pst and FAW, key physiological and metabolomic findings, and their significance.

The abstract has now been thinned and summarizes the overall work rather than providing details. The abstract has been reduced from 300 words to 244.

The introduction provides good context but could better justify why Pst was chosen for potato, given its primary association with tomato and Arabidopsis. Strengthen the rationale for studying Pst on potato by citing evidence of its cross-host infectivity (e.g., Arabidopsis, tomato) and clarifying the knowledge gap.

When pressure infiltration is used as a means to inoculate the plant the physical barriers are overcome and this induces a response in any plant. In that case established host-parasite relationships count less, and therefore it matters less if the bacterium studied is a known pathogen of the crop on which it is tested. For the present study we used pressure infiltration as a means to inoculate the plants, and this must have given Pst an advantage since it did not have to overcome physical barriers. Furthermore, Pst has a host range which includes Arabidopsis, tobacco, olive, and green bean and therefore we reasoned that the defense of potato which belongs to the same family as tomato would easily be overcome. Moreover, Pst is a “model” for studying plant-pathogen interactions because of its well-studied type III secretion system and using Pst on potato allows one to compare potato and tomato with ease. This explanation has now been included.

Specify the potato cultivar’s susceptibility to FAW and Pst to justify its selection. –

No potato resistances to the two parasites, FAW and Pst, are mentioned for the chosen cultivar, Georgina. The websites <https://www.potatopro.com/> and <https://potatoes.agricrops.org/> have the cultivar information. This variety was chosen for its popularity. Many other varieties would have been chosen with our criteria of popularity. This explanation is now included in the manuscript.

Clarify the timing of metabolomic sampling (e.g., were leaves sampled at day 63, as implied by Figs 4- 9?). –

The interest was to look at permanent changes in the metabolomic profile. Studies which investigate direct responses to infection grab the window of within 72 hours, or a little longer, post inoculation/infestation. In such cases the metabolites of interests are those which respond to overcome infestation and infection. In the case of this current work, sampling within 72 hours would disturb pest feeding and the larvae would probably escape from the caging. Since our interest was in permanent metabolomic changes, we resorted to doing the metabolomic analysis at termination, 63 days after parasite exposure. Metabolomic analysis at this time point proved valuable because the experimental treatments could be still be differentiated. Ideally, multiple points of sampling would have provided a clearer picture of the metabolomics of plant-parasite relations.

Provide more details on statistical analyses (e.g., assumptions for ANOVA, handling of non-normal data).

I have now included that ANOVA was used for all the growth and gas exchange data. Analysis of the metabolomics data required the chemometrics tools of principal component analysis, partial least squares discriminant analysis, sparse PLS-DA and orthogonal PLS-DA.

The results section is overly descriptive, repeating data from tables and figures without synthesizing key trends. For example, the discussion of physiological parameters could be more concise, focusing on significant differences and their biological implications. Avoid repeating table/figure data verbatim; instead, highlight significant trends and refer to visuals. Resolve the discrepancy between Figure 3 and text regarding tuber weight significance.

This has now been handled and it appear red in the corrected version.

The discussion is comprehensive but includes speculative statements (e.g., “the bacterium deters the pest”) without sufficient mechanistic evidence. The overlap in metabolomic profiles between FAW+Pst and control treatments (Fig. 9) is noted but not fully explored.

The bacterium probably deterring the pest was an accidental finding in this current work. I have written that it requires further investigation. The statement that the bacterium deters the pest comes from the fact that the ability of the pest to reduce plant height lessened in the presence of the pest. Some strains of the genus Pseudomanas like Pseudomonas fluorescens have these antagonistic properties against parasites. It could be this property which was activated in the presence of the pest. I have included this explanation although it is further speculation.

Tables 1 and 2 are informative but could be combined into a single table with subheadings for 13-day and 34- day measurements to save space and improve

This has been done.

Figure 3 states “no statistically significant differences” in tuber weight, which contradicts the text (Page 24, Line 291) claiming significant reductions by individual parasites. This discrepancy needs resolution.

This has now been resolved and it appears red in the corrected manuscript.

Overstatement of Pst deterrence: - The claim that Pst deters FAW is based primarily on the lack of plant height reduction in co-treated plants and the overlap in metabolomic profiles. However, alternative explanations (e.g., compensatory plant responses, reduced FAW feeding due to plant stress) are not considered. - Metabolomic Insights: - The discussion of metabolites like α-solanine, 6- gingerol, and rutin is interesting but lacks depth. For example, the downregulation of α-solanine is noted, but its potential role in plant defense or FAW deterrence is not explored. Expand the discussion of key metabolites, linking their changes to known defense pathways or pest/pathogen interactions. For instance, discuss whether rutin downregulation aligns with Pst’s manipulation of plant defenses (as hinted in López-Gresa et al., 2011).

Because some members of the genus Pseudomonas, such as *Pseudomonas flourescens*, have antagonistic properties against parasites. It could be that this property was activated in the presence of the pest, bearing in mind that this combination of Pst and the fall armyworm has never been explored. This is pure speculation and it has been mentioned as such in the corrected version of the manuscript. I also mentioned that this aspect requires further exploration. The overlap in metabolomic profiles as revealed on Figure 9 aligns well with this parasite-to-parasite cancellation effect proposition. This was an accidental discovery in this manuscript and it never formed part of the objectives which were assessing the effect on the parasites on the plants but not on each other. I agree with the reviewer that compensatory plant responses, reduced FAW feeding due to plant stress could have caused this “appear to be deterring” phenomenon. If our metabolomic analysis was within 72 hours of infestation and inoculation we could have seen a different spectrum of metabolomic upregulation, and probably rutin would have been upregulated and we would easily implicate known defense pathways like the phenylpropanoid pathway. Metabolomic analysis done at 63 days post infestation is a different matter altogether, it is unique in this study. Although we are able to attribute the metabolomic changes to infestation and inoculation, we are unable to prove that the metabolomic reprogramming we observed was for defense.

The manuscript concludes that the findings add valuable knowledge, but it does not discuss practical implications for potato farming or pest management (e.g., could Pst or related bacteria be used as biocontrol agents against FAW?). Minor Issues - Typographical and grammatical errors: - There are minor errors, e.g., “ineral ratio” (Page 1, Abstract) should be “Ci/Ca ratio,” and “uninoculated” is inconsistently hyphenated (e.g., Pages 20-22). Conduct a thorough proofread to correct typos and ensure consistency in terminology.

This is now handled, and it is corrected in the current manuscript. All minor errors including on the figures and tables have been sorted.

**Reviewer 2**

Terms like “Ci/Ca” are used without explanation.

The full meaning is written in blue font.

The enumeration of effects does not follow a logical order.

The abstract has been reorganized. The affected part has been written in blue.

“Combined with the bacterium…” is repeated several times.

I have changed some to co-administered and joint FAW-*Pseudomonas syringae* to lessen the number of times of the “combined”. The font is changed to blue.

Enter a financial disclosure statement that describes the sources of funding for the work included in this submission. Review the submission guidelines for detailed requirements. View published research articles from PLOS ONE for specific examples. Funded studies. Enter a statement with the following details: Initials of the authors who received each award. Grant numbers awarded to each author. The full name of each funder. URL of each funder website. Did the sponsors or funders play any role in the study design, data collection and analysis, decision to publish, or preparation of the manuscript?

This statement now reads: This study was partially funded by the National Research Foundation of South Africa (www.nrf.ac.za) under the grant TTK170413227119. The funds were allocated to Khayalethu Ntushelo and this research was covered solely by his funds. The funder did not take part in the study design and in any aspect of the study. Blue font to show the changes.

Too much information about FAW:

The section on the nocturnal behavior of the two FAW biotypes is unnecessary. Suggestion: Reduce to a concise paragraph.

This part was removed: These strains have different timings for mating activities. For the C-strain, female calling and mating occur early in the night and for the R-strain, it happens during the last half of the night.

Include a logical transition between the description of the pathogen and the motivation for the study.

This part was included: The plethora of information on *P. syringae* pv*. tomato* has left this critical knowledge gap whose closure can augment our understanding of potato plant responses to parasitism. It is also important not to study one aspect of response to parasitism but the entire package of growth, physiology and metabolomics. See blue font.

Lack of clearly described statistical replication: Example: “n = 4” appears in tables, but is not explained in the text. Specify the number of replicates per treatment. Technical use without methodological reference: The LC-MS technique could be better substantiated with references.

Inconsistency in collection time: Collection time varies, but this is not discussed in terms of its impact on results. Ambiguous description of the experimental design: Include a clearer description of the statistical design (e.g. factorial, randomized blocks).

Replication is now included in the text. For the references of the LC-MS technique I have added Ramabulana et al. [14] as the methods were a modification from this article. On the the trial layout, it was complete randomization since the glasshouse conditions are homogenous. I have added that ANOVA was used to analyze the data. Look for the sentence: Data was analyzed by using analysis of variance aided with the use of the software STATISTICA version 10; significant differences (p = 0.05) were determined using the Duncan multiple range test to separate the means of the measured parameters. On the inconsistency in collection time: Seven-day intervals were chosen from day 13 post-parasite exposure. We were careful not to disturb the pest on the first 13 days and from day 13 we selected days 20, 27 and 34 as data capture points, and this was consistent with our pre-determined seven-day interval between data capture points. From previous unpublished work we observed that growth and physiology responses take place during the period selected in the current study. Harvest at day 63 was the point at which tubers had formed, and it was not pre-determined but was decided after observing foliage changes.

This explanation has now been included, see blue font, and surely a very good point by the reviewer.

Little discussion of how the results align or contrast with previous studies. It could include practical implications or suggestions for integrated management.

There is paucity of results on this aspect of this study. This must be because of its uniqueness. References 24, 27, 28, 41, 44, 45, 52, 61, 63, 65, 66, 67 and 68 are the only ones closest and that is why our discussion was limited only to them. However, we have added a paragraph on implications for integrated management. The paragraph reads:

Despite the inconclusive aspects of this study, the results obtained are valuable and add crucial knowledge to our understanding of potato-parasitism dynamics. Should the deterrent of the pest by the bacterium be confirmed, a potential biological control product is possible, and this may be a benefit to potato farmers if the FAW becomes devastating. Moreover, given that some parameters correlated strongly with others it is possible to manipulate one parameter to affect another parameter. For an example, total tuber weight depends strongly on plant height and stem diameter. This means a grower may aim for taller and thicker plants to boost yield. Additionally, the number of tubers per plant also depends on water use efficiency which means any agent which boosts water use efficiency may affect this important yield parameter, the number of tubers per plant. As various efforts of plant manipulation continue, optimal and best practice combinations will be discovered and this will improve potato productivity. This study helped to confirm the interlinks between plant processes for better understanding of plant processes, especially plants exposed to parasites.

Yours sincerely,

Prof. Khayalethu Ntushelo

Signed:
